# Analysis of the correlation and influencing factors between delirium, sleep, self-efficacy, anxiety, and depression in patients with traumatic brain injury: a cohort study

**DOI:** 10.3389/fnins.2024.1484777

**Published:** 2024-11-01

**Authors:** Zhongmin Fu, Xiaoju Miao, Xian Luo, Lili Yuan, Yan Xie, Shiming Huang

**Affiliations:** ^1^Department of Nursing, Affiliated Hospital of Zunyi Medical University, Zunyi, China; ^2^The First Ward of the Neurosurgery Department, Affiliated Hospital of Zunyi Medical University, Zunyi, China; ^3^Department of Nursing, West China Hospital of Sichuan University, Chengdu, China

**Keywords:** traumatic brain injury, anxiety, depression, delirium, sleep, self-efficacy

## Abstract

**Background:**

Patients with traumatic brain injury (TBI) often experience post-injury anxiety and depression, which can persist over time. However, the relationships between anxiety and depression in TBI patients and delirium, sleep quality, self-efficacy, and serum inflammatory markers require further investigation.

**Objective:**

This study aims to explore the associations of delirium, sleep quality, self-efficacy, and serum inflammatory markers with anxiety and depression in TBI patients, and to examine potential influencing factors.

**Methods:**

We conducted a cohort study involving 127 patients with TBI. Delirium was assessed using the Confusion Assessment Method (CAM) and CAM-ICU, while anxiety, depression, sleep quality, self-efficacy, and pain were evaluated using the appropriate tools, respectively. Serum inflammatory markers (CRP, TNF-α, IL-6) were collected within 1 day post-injury. Generalized estimating equations (GEE) were used to analyze the relationships between delirium, sleep, self-efficacy, and anxiety/depression.

**Results:**

The study identified 56 patients with delirium. Patients with delirium differed significantly from those without delirium in age, TBI classification, sleep duration, CRP levels, TNF-α levels, pain, self-efficacy, and insomnia (*P* < 0.05). The GEE analysis revealed that delirium, CRP levels, self-efficacy, underlying diseases, insomnia, TBI classification, age, and sleep duration were associated with anxiety symptoms in TBI patients at 6 months post-discharge (*P* < 0.05). Depression in TBI patients at 6 months post-discharge was not associated with delirium or insomnia but correlated with CRP levels, TBI classification, and self-efficacy (*P* < 0.05).

**Conclusion:**

TBI patients who experience delirium, insomnia, and low self-efficacy during the acute phase are likely to exhibit more anxiety at the 6-month follow-up. Depression in TBI patients is not associated with delirium or insomnia but is negatively correlated with self-efficacy. CRP levels post-TBI may serve as a biomarker to identify patients at risk of emotional symptoms and potentially accelerate patient recovery.

## 1 Introduction

Traumatic brain injury (TBI) is a significant cause of disability and mortality worldwide. It is estimated that ~10 million people globally experience TBI annually, with 52,000 deaths attributed to TBI and nearly 100,000 new cases of disability resulting from it (Ahmed et al., 2017; Capizzi et al., 2020l Hyder et al., 2007). TBI can lead to a spectrum of cognitive, social, emotional, physical, and behavioral impairments, including sleep disturbances, depression/anxiety, and cognitive deficits (Draper and Ponsford, 2008; Langlois et al., 2006; Morganti-Kossmann et al., 2019; Dikmen et al., 2009). Studies have reported prevalence rates of anxiety disorders among TBI patients ranging from 21 to 70% (Moore et al., 2006; Scholten et al., 2016), and depression rates ranging from 17 to 50% (Scholten et al., 2016; Riggio, 2011; Osborn et al., 2014). These mental health issues not only significantly reduce patients' quality of life but also potentially impact their recovery process and social functioning (Duan et al., 2021; Rapoport et al., 2006; Zahniser et al., 2019). Given the complex etiology of psychological manifestations in TBI patients, which involve a combination of brain dysfunction and psychological trauma, as well as interrelationships between cognitive, emotional, and physical symptoms, epidemiology and underlying mechanisms of psychological health in TBI patients remain areas worthy of exploration (Piantino et al., 2022; Brett et al., 2022; Dikmen et al., 2009).

Delirium is characterized by an acute alteration in mental status marked by confusion, impaired attention, and fluctuating levels of arousal. Trauma-induced agitation and delirium generally manifest within the initial 24 h of TBI admission but can occur at any point during hospitalization and are frequent during the recovery phase (Roberson et al., 2021). Prior research indicates that patients experiencing trauma-induced delirium are susceptible to severe acute and long-term psychiatric outcomes (Teasdale and Jennett, 1976; Draper et al., 2007; Rocca et al., 2019). However, there is currently no direct investigation into the correlation between trauma-induced delirium in TBI patients and anxiety or depression during the recovery period.

Insomnia is highly prevalent among individuals with TBI, affecting 29% of patients (Mathias and Alvaro, 2012). Within 10 days post-TBI, about 13.3% of patients report sleep disturbances, increasing to 33.5% within 6 weeks (Chaput et al., 2009). Chaput et al. (2009) investigated sleep complaints in 493 mild TBI patients at 10 days and 6 weeks post-injury, revealing that those with sleep issues faced heightened risks of headaches, depressive symptoms, and irritability. Moreover, TBI patients experiencing sleep problems were more likely to concurrently suffer from depressive symptoms (Chaput et al., 2009), and prolonged insomnia has been linked with worsening depression over time (Lequerica et al., 2020; Huang et al., 2013). Nonetheless, there remains limited longitudinal and temporal research on the interplay between sleep quality, depression, and anxiety (Saravanan et al., 2024; Rao et al., 2014), highlighting the need for additional prospective studies to clarify the causal relationship between acute insomnia during TBI and the heightened occurrence of post-injury depression and anxiety symptoms.

Self-efficacy refers to one's confidence in managing symptoms, indicating greater persistence in related tasks (Bandura, 1977). In acquired brain injury, higher self-efficacy is associated with reduced anxiety and depression symptoms (Lewin et al., 2013; Longworth et al., 2018; Volz et al., 2016; Brands et al., 2015). Studies indicate that patients with higher self-efficacy, goal resilience, and adaptive goal adjustment tend to experience fewer emotional disturbances post-injury (Brands et al., 2015). Improving self-efficacy levels may be crucial for addressing psychological distress in mild TBI patients (Belanger et al., 2020). However, research on the relationship between self-efficacy and anxiety/depression has predominantly focused on stroke patients with acquired brain injury, with limited exploration in TBI patients.

Inflammation is a significant secondary mechanism following TBI (Das et al., 2012), potentially linked to various neurological symptoms such as anxiety, depression, cognitive impairment, and sleep disturbances (Malik et al., 2022; Rathbone et al., 2015; McAfoose and Baune, 2009). Research suggests interleukin-6 (IL-6), tumor necrosis factor α (TNFα), and C-reactive protein (CRP) are the predominant cytokines associated with adverse psychological outcomes in mild TBI patients (Malik et al., 2022; Rathbone et al., 2015). Additionally, systemic inflammation appears to correlate with psychological conditions like depression and anxiety in non-TBI populations (Osimo et al., 2020; Silva-Fernandes et al., 2024). However, it remains unclear whether these inflammatory mediators exhibit heightened activity in individuals experiencing post-TBI depression and anxiety. Discrepancies in the timing of cytokine assessments post-injury were noted in a systematic review, with most studies evaluating participants during the chronic phase of TBI (ranging from 1 month to several years) (Malik et al., 2022). Currently, the relationship between elevated acute-phase serum inflammatory markers in TBI and increased emotional symptoms requires further investigation.

This study aims to explore relationships between anxiety and depression symptoms, delirium, sleep, self-efficacy, and acute-phase serum inflammatory markers in TBI patients through a prospective cohort study. Findings are expected to deepen understanding of anxiety and depression mechanisms post-TBI, support future clinical interventions, and enhance long-term recovery and quality of life for patients.

## 2 Methods

### 2.1 Study design

This is a 6-month prospective cohort study approved by the Medical Ethics Committee of Affiliated Hospital of Zunyi Medical University. The study adheres to the Helsinki Declaration (Goodyear et al., 2007) and follows the Strengthening the Reporting of Observational Studies in Epidemiology (STROBE) guidelines (von Elm et al., 2007).

### 2.2 Study setting

This study was conducted at the Neurosurgery Ward of the Affiliated Hospital of Zunyi Medical University, a tertiary hospital in Guizhou Province, China. The study commenced in June 2022 and concluded in December 2023. Patient recruitment began upon admission from June 2022 to April 2023. A research assistant (LX) explained the study objectives to patients and their families, ensuring informed consent was obtained and documented. Follow-up was conducted by designated nurses (FZM), starting from the discharge of the first patient until 6 months post-discharge, with follow-up intervals at 1-, 3-, and 6-months post-discharge.

### 2.3 Study population

This study is a prospective, population-based cohort study focusing on patients with TBI. Convenience sampling was employed to select patients from the Neurosurgery Ward of the Affiliated Hospital of Zunyi Medical University from June 2022 to April 2023. Inclusion criteria: confirmed history of TBI using CT or MRI; patients discharged from the hospital; stable vital signs at discharge. Exclusion criteria: severe endocrine and metabolic diseases, hematologic disorders, malignant tumors, chronic pulmonary insufficiency, liver or kidney failure; patients who died within 3 days post-injury; inability to participate in follow-up due to speech impediment or refusal.; history of previous TBI, stroke, brain tumors, epilepsy, cognitive impairments or other brain disorders. Chronic inflammation associated with these comorbidities could distort the study results. Excluding these patients helps reduce confounding factors, enabling a more accurate analysis of the relationship between post-traumatic brain injury inflammation and clinical outcomes.

### 2.4 Data collection

#### 2.4.1 Basic information

Basic information was registered via the hospital information system from patient admission to discharge. This included baseline socio-demographic characteristics and medical history, encompassing age, gender (male and female), residential types, marital status, education, underlying diseases, body mass index (BMI), monthly family income, and TBI classification. Underlying diseases refer to pre-existing conditions upon admission, such as hypertension, diabetes, and chronic obstructive pulmonary disease. TBI was classified based on the Glasgow Coma Scale (GCS) score at admission, with scores of 13–15 indicating mild TBI, 9–12 indicating moderate TBI, and 3–8 indicating severe TBI (Rimel et al., 1982).

#### 2.4.2 Measurement of serum CRP, TNF-α, and IL-6

Fasting venous blood samples (3 mL) were collected from patients on the morning of the first post-injury day in citrate anticoagulant tubes. After centrifugation at 3,000 rpm for 15 min, the supernatant was stored at −20°C until analysis. Serum CRP, TNF-α, and IL-6 levels were determined using the ELISA sandwich method. Experimental procedures adhered strictly to the manufacturer's instructions.

#### 2.4.3 Pain

Pain assessment was conducted within 1-day post-injury using the critical-care pain observation tool (Gélinas et al., 2006). This tool evaluates four dimensions: facial expression, body movements, muscle tension, and compliance with ventilator synchrony or vocalization. The dimension “ventilator synchrony” applies to intubated patients, while “vocalization” applies to non-intubated patients. Each dimension is scored from 0 to 2, yielding a total score of 8 points. A score of 0 indicates no pain, while 8 indicates maximum pain. Evaluation was performed by physicians.

#### 2.4.4 Sleep duration

On the day of discharge, nurses investigated patients' self-reported sleep duration by asking, “How many hours did you sleep each night in the past week ?”

#### 2.4.5 Sleep quality

The Athens Insomnia Scale (AIS) was employed to assess patient sleep quality scores. This assessment was conducted upon discharge. The AIS comprises eight dimensions: sleep onset latency (time from lights out to falling asleep), nocturnal awakenings, early morning awakenings before desired time, total sleep time, overall sleep quality (irrespective of duration), daytime mood disturbance, daytime physical functioning (physical or mental, e.g., memory, cognition, attention), and daytime sleepiness (Soldatos et al., 2000). Each dimension is rated from 0 (no impact) to 3 (severe impact), with a total score of 24. Higher scores indicate poorer sleep quality. Scores below 4 indicate no insomnia, 4–6 suggest suspected insomnia, and scores above 6 indicate insomnia.

#### 2.4.6 Self efficacy

Schwarzer et al. (2009) developed the general self-efficacy scale (GSES) to evaluate how individuals cope in different situations and their confidence facing new challenges. The scale comprises 10 items rated on a 4-point Likert scale, ranging from 1 (completely incorrect) to 4 (completely correct). The final score is obtained by summing the scores of all items and dividing by 10. A higher score indicates greater self-efficacy in patients. Scores between 1.0 and 2.0 suggest low self-efficacy, scores between 2.1 and 3.0 indicate moderate self-efficacy, and scores between 3.1 and 4.0 indicate high levels of self-efficacy. Assessment using the GSES was conducted at the patients' discharge.

#### 2.4.7 Delirium

Delirium assessment was conducted using the confusion assessment method (CAM) (Marcantonio et al., 2014) or the CAM-ICU (Ely et al., 2001) by responsible nurses and physicians at 8:00 a.m. and 8:00 p.m. daily for target patients post-injury. If any assessment during hospitalization met the delirium criteria, the patient was considered to have developed delirium. All nurses and physicians were trained in using delirium assessment tools to ensure accuracy. CAM evaluates delirium based on four features corresponding to four specific question items: (1) acute onset or fluctuating course of mental status; (2) inattention; (3) disorganized thinking; (4) altered level of consciousness. The CAM diagnostic algorithm requires the simultaneous presence of criteria (1) and (2), and either criterion (3) or (4). For patients in the ICU or those unable to communicate verbally due to endotracheal intubation, the CAM-ICU was used to assess delirium. When using this scale, initial assessment of sedation depth was performed using the Richmond agitation-sedation scale (RASS) (Ely et al., 2003). Delirium assessment was conducted if the RASS score was −3 or higher. A RASS score below −3 indicated the patient was unconscious, in which case delirium assessment was temporarily suspended.

#### 2.4.8 Anxiety and depression assessment

Psychological status among patients was evaluated using the hospital anxiety and depression scale (HADS) at discharge and during follow-ups at 1-, 3-, and 6-months post-discharge. We selected discharge time as the baseline since it marks a stable recovery phase and the end of primary treatment, enhancing comparability. Developed by Zigmond and Snaith (1983) in 1983, the HADS comprises two subscales: anxiety (HADS-A) and depression (HADS-D). Each subscale comprises seven items, each scored from 0 to 3 points, resulting in a maximum subscale score of 21 points, with higher scores indicating more severe psychological distress. The scores of 0–7 indicate normal psychological status, 8–10 suggest mild anxiety or depression, 11–14 indicate moderate levels, and scores between 15 and 21 signify severe anxiety or depression. These assessments provide valuable insights into the patients' mental health over time, aiding in appropriate intervention and support strategies.

### 2.5 Sample size

The sample size was calculated using PASS 15 software based on the longitudinal data sample size formula provided by Diggle (2002). The primary outcome was the HADS-A score at four time points. A pilot study of 30 patients (15 in the delirium group and 15 in the non-delirium group) showed an expected mean difference in anxiety scores of 6.2, with a pooled standard deviation of 3.87 and a repeated measures correlation of 0.812. Considering a two-sided significance level of 0.05, a power of 0.9, and a 20% dropout rate, the sample size was calculated to be 20. Ultimately, 130 patients were enrolled, with 127 completing follow-up.

### 2.6 Statistical analysis

Data were exported using Excel 2016 and reviewed by two individuals for accuracy. Statistical analyses were conducted using STATA 17 software. Continuous variables following a normal distribution were described using mean and standard deviation (SD). Between-group comparisons were assessed using *t*-tests. Non-normally distributed continuous variables were described using median (M) and interquartile range (IQR), with between-group comparisons evaluated using the Wilcoxon rank-sum test. Categorical variables were described as frequencies and percentages, with between-group comparisons analyzed using the chi-square test. Given the repeated measures of anxiety and depression scores among TBI patients and potential missing data, the generalized estimating equation (GEE) model was used with the maximum likelihood estimation method. As the missing data was < 5%, no sensitivity analysis was conducted (Schafer, 1997). A preliminary normality test of the HADS scores was performed before the GEE analysis. Since the scores followed a normal distribution, a Gaussian distribution with an identity link function was chosen. Dummy variables were used in the GEE model to identify factors influencing anxiety and depression. Further analyses using the GEE model compared HADS-A scores between delirium and non-delirium groups, non-insomnia, suspected insomnia and insomnia groups, and low, medium, and high self-efficacy groups at different time points relative to discharge day. Graphs were created using GraphPad Prism 9.0. A significance level of α = 0.05 (two-sided) was used, with *P* < 0.05 indicating statistical significance.

## 3 Results

### 3.1 Comparison of baseline characteristics between delirium and non-delirium groups in TBI patients

**Figure 1:**
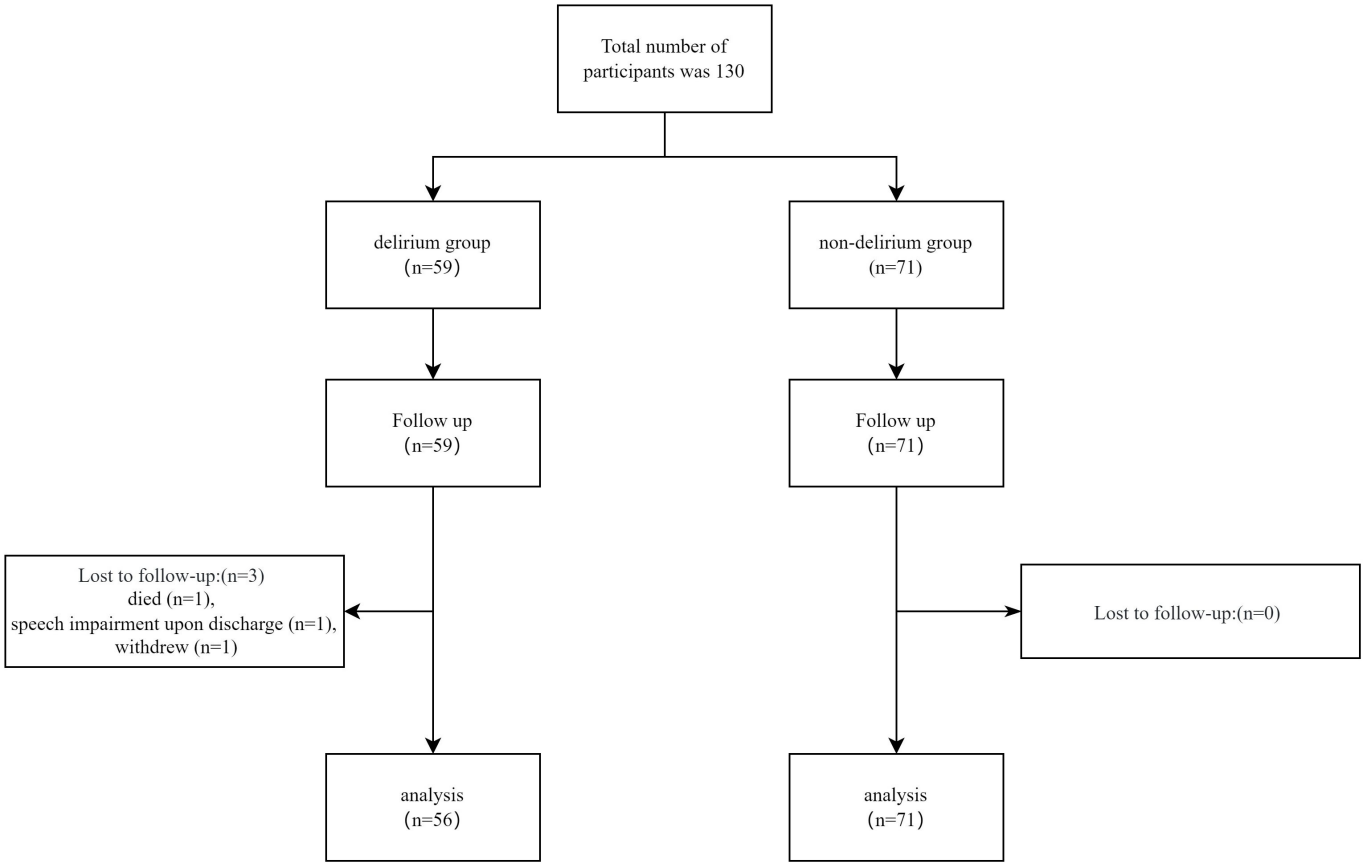
Flowchart of participant enrollment.

A total of 130 TBI patients were recruited for this study, with three patients lost to follow-up: one patient died, one patient developed speech impairment making communication impossible upon discharge, and one patient withdrew. Ultimately, data from 127 patients were included in the final analysis. Details of patient recruitment and follow-up are illustrated in Figure 1. Among these 127 TBI patients, 56 (44.1%) experienced delirium. General characteristics of all TBI patients and differences between the delirium and non-delirium groups are summarized in Table 1. Significant differences between the delirium and non-delirium groups were observed in terms of age, TBI classification, sleep duration, CRP levels, TNFα levels, pain scores, self-efficacy, and insomnias (*P* < 0.05, Table 1).

**Table 1:** General characteristics of delirium and non-delirium groups in patients with traumatic brain injury.

**Variable**		**Non-delirium group (*n* = 71)**	**Delirium group (*n* = 56)**	**χ^2^/*t*/*Z***	***P*-value**
Gender, *n* (%)	Man	49 (69.0)	36 (64.3)	0.316	0.574
	Woman	22 (31.0%)	20 (35.7%)		
Age, *n* (%)	18–30	4 (7.2)	14 (19.7)	14.374	0.001
	31–59	27 (48.2)	11 (15.5)		
	≥60	25 (44.6)	46 (64.8)		
Residential types, *n* (%)	Rural	23 (32.4)	18 (32.1)	0.001	0.976
	Urban	48 (67.6)	38 (67.9)		
Marital status, *n* (%)	Married	43 (60.6)	34 (60.7)	0.001	0.986
	Single	28 (39.4)	22 (39.3)		
Work status, *n* (%)	Employed	43 (60.6)	30 (46.4)	0.626	0.429
	Unemployed	28 (39.4)	26 (53.6)		
Education, *n* (%)	Primary school and below	10 (14.1)	8 (14.3)	0.703	0.951
	Middle school	18 (25.3)	11 (19.6)		
	High school	22 (31.0)	20 (35.7)		
	Junior college	13 (18.3)	10 (17.9)		
	Bachelor and above education degree	8 (11.3)	7 (12.5)		
Underlaying disease, *n* (%)	No	33 (46.5)	18 (32.1)	2.677	0.102
	Yes	38 (53.5)	38 (67.9)		
BMI (kg/m^2^), *n* (%)	< 18.5	3 (4.3)	2 (3.6)	0.645	0.886
	18.5–23.9	26 (36.6)	17 (30.4)		
	24–27.9	28 (39.4)	25 (44.6)		
	≥28	14 (19.7)	12 (21.4)		
Monthly family income (yuan), *n* (%)	< 5,000	8 (11.3)	4 (7.1)	0.937	0.626
	5,000–10,000	27 (38.0)	25 (44.7)		
	>10,000	36 (50.7)	27 (48.2)		
TBI classification, *n* (%)	Mild TBI	47 (66.2)	2 (3.6)	51.816	< 0.001
	Moderate TBI	24 (33.8)	54 (96.4)		
Sleep duration (min)		308.82 ± 50.56	282.47 ± 51.31	2.897	0.004
CRP (mg/L)		25.18 (14.91)	44.80 (12.07)	−5.189	< 0.001
TNFα (pg/ml)		173.91 (38.55)	268.74 (51.99)	−6.517	< 0.001
IL6 (pg/ml)		285.34 (214.81)	288.67 (222.44)	−1.513	0.13
CPOT score		3.15 ± 1.54	4.53 ± 1.29	−5.386	< 0.001
AIS		11.31 ± 5.87	13.41 ± 4.41	−2.225	0.028
GSES		2.81 ± 1.26	1.74 ± 0.49	5.961	< 0.001

TBI, traumatic brain injury; GSES, general self-efficacy scale; CPOT, critical-care pain observation tool; AIS, Athens insomnia scale; CRP, C-reactive protein; TNF α, tumor necrosis factor α; IL-6, interleukin-6.

### 3.2 Factors influencing anxiety in patients with TBI

Using HADS-A scores as the dependent variable, the GEE analysis revealed that delirium, CRP levels, self-efficacy, underlaying disease, insomnias, TBI classification, age, and sleep duration were significant factors influencing anxiety in traumatic brain injury patients post-discharge (*P* < 0.05, Table 2).

**Table 2:** Parameter estimation of anxiety factors based on generalized estimation equation.

**Parameter**	**β**	**SE**	**95%CI**		**Waldχ^2^**	***P*-value**
			**Lower limit**	**Upper limit**		
Intercept	5.804	2.0171	1.851	9.758	8.281	0.004
**Time**						
6 months	−3.071	0.1206	−3.307	−2.835	648.647	0.000
3 months	−2.159	0.1008	−2.357	−1.962	458.898	0.000
1 months	−0.429	0.1021	−0.630	−0.229	17.670	0.000
Baseline	0^a^	–	–	–	–	–
**Gender**						
Female	0.208	0.2673	−0.316	0.732	0.605	0.437
Male	0^a^	–	–	–	–	–
**Age**						
18–39 y	1.211	0.3686	0.488	1.933	10.790	0.001
40–59 y	0.532	0.3674	−0.188	1.253	2.099	0.147
≥60 y	0^a^	–	–	–	–	–
**Residential types**						
Rural	0.029	0.3519	−0.661	0.718	0.007	0.935
Urban	0^a^	–	–	–	–	–
**Marital status**						
Single	0.340	0.3097	−0.267	0.947	1.203	0.273
Married	0^a^	–	–	–	–	–
**Work status**						
Unemployed	−0.138	0.2559	−0.640	0.363	0.292	0.589
Employed	0^a^	–	–	–	–	–
**Education**						
Bachelor and above education degree	0.232	0.5140	−0.775	1.240	0.204	0.652
Junior college	0.188	0.5416	−0.873	1.250	0.121	0.728
High school	−0.225	0.4382	−1.083	0.634	0.263	0.608
Middle school	−0.218	0.4727	−1.144	0.709	0.212	0.645
Primary school and below	0^a^	–	–	–	–	–
**Underlaying disease**						
Existence	0.779	0.2583	0.272	1.285	9.089	0.003
Non-existence	0^a^	–	–	–	–	–
**BMI (kg/m** ^2^ **)**						
≥28	0.022	0.9239	−1.789	1.833	0.001	0.981
24–27.9	0.414	0.9566	−1.461	2.289	0.187	0.665
18.5–23.9	0.252	0.9136	−1.538	2.043	0.076	0.782
< 18.5	0^a^	–	–	–	–	–
**Monthly family income (yuan)**						
>10,000	−0.943	0.5740	−2.068	0.182	2.697	0.101
5,000–10,000	−0.715	0.6187	−1.928	0.497	1.337	0.248
< 5,000	0^a^	–	–	–	–	–
**TBI classification**						
Mild TBI	1.508	0.3535	0.815	2.201	18.208	0.000
Moderate TBI	0^a^	–	–	–	–	–
**Delirium**						
Yes	1.148	0.3866	0.390	1.906	8.819	0.003
No	0^a^	–	–	–	–	–
**AIS score**						
>6	0.953	0.4874	−0.002	1.909	3.826	0.050
4–6	0.366	0.4345	−0.486	1.218	0.709	0.400
0–4	0^a^	–	–	–	–	–
**GSES score**						
5–6	−1.076	0.4006	−1.861	−0.291	7.219	0.007
3–4	−0.093	0.2941	−0.669	0.484	0.099	0.753
1–2	0^a^	–	–	–	–	–
CPOT score	−0.063	0.1084	−0.276	0.149	0.340	0.560
CRP (mg/L)	0.057	0.0156	0.026	0.087	13.258	0.000
TNFα (pg/ml)	0.001	0.0033	−0.007	0.006	0.019	0.889
IL−6 (pg/ml)	0.002	0.0011	0.000	0.004	2.536	0.111
Sleep duration (min)	−0.020	0.0097	−0.039	−0.001	4.110	0.043

TBI, traumatic brain injury; GSES, general self-efficacy scale; CPOT, critical-care pain observation tool; AIS, Athens insomnia scale; CRP, C-reactive protein; TNF α, tumor necrosis factor α; IL-6, Interleukin-6.

0^a^ refers to the redundant parameter; β refers to the regression coefficient or model parameter; “–” indicates that the group is the reference group.

### 3.3 Factors influencing depression in patients with TBI

Using HADS-D scores as the dependent variable, the GEE analysis revealed that post-discharge depression in traumatic brain injury patients was not associated with delirium, insomnias but showed significant correlations with CRP levels, TBI classification, and self-efficacy (*P* < 0.05, Table 3).

**Table 3:** Parameter estimation of depression factors based on generalized estimation equation.

**Parameter**	**β**	**SE**	**95%CI**		**Waldχ^2^**	***P*-value**
			**Lower limit**	**Upper limit**		
Intercept	6.775	2.7695	1.347	12.203	5.984	0.014
**Time**						
6 months	−3.537	0.1596	−3.850	−3.224	490.878	0.000
3 months	−3.007	0.2125	−3.423	−2.591	200.324	0.000
1 months	−0.587	0.1265	−0.835	−0.339	21.542	0.000
Baseline	0^a^	–	–	–	–	–
**Gender**						
Female	0.105	0.3644	−0.609	0.819	0.083	0.773
Male	0^a^					
**Age**						
18–39 y	0.540	0.4601	−0.362	1.441	1.376	0.241
40–59 y	0.035	0.4394	−0.826	0.896	0.006	0.937
≥60 y	0^a^	–	–	–	–	–
**Residential types**						
Rural	−0.562	0.3352	−1.219	0.095	2.808	0.094
Urban	0^a^	–	–	–	–	–
**Marital status**						
Single	0.327	0.3428	−0.345	0.999	0.910	0.340
Married	0^a^	–	–	–	–	–
**Work status**						
Unemployed	−0.244	0.2878	−0.808	0.320	0.718	0.397
Employed	0^a^	–	–	–	–	–
**Education**						
Bachelor and above education degree	−0.331	0.5938	−1.495	0.832	0.311	0.577
Junior college	−0.038	0.6204	−1.254	1.178	0.004	0.951
High school	−0.151	0.5158	−1.161	0.860	0.085	0.770
Middle school	−0.015	0.4563	−0.909	0.880	0.001	0.975
Primary school and below	0^a^	–	–	–	–	–
**Underlaying disease**, ***n*** **(%)**						
Existence	0.393	0.2716	−0.139	0.925	2.093	0.148
Non-existence	0^a^	–	–	–	–	–
**BMI (kg/m** ^2^ **)**						
≥28	−1.600	1.2553	−4.060	0.860	1.625	0.202
24–27.9	−1.792	1.2621	−4.266	0.681	2.017	0.156
18.5–23.9	−1.412	1.2554	−3.873	1.048	1.266	0.261
< 18.5	0^a^	–	–	–	–	–
**Monthly family income (yuan)**						
>10,000	−0.575	0.5038	−1.563	0.412	1.304	0.253
5,000–10,000	−0.752	0.5534	−1.836	0.333	1.844	0.174
< 5,000	0^a^	–	–	–	–	–
**TBI classification**						
Mild TBI	0.922	0.3862	0.165	1.679	5.695	0.017
Moderate TBI	0^a^	–	–	–	–	–
**Delirium**						
Yes	0.222	0.4522	−0.665	1.108	0.240	0.624
No	0a	–	–	–	–	–
**AIS score**						
>6	0.658	0.5830	−0.485	1.800	1.272	0.259
4–6	0.499	0.4871	−0.456	1.453	1.048	0.306
0–4	0^a^	–	–	–	–	–
**GSES score**						
5–6	−0.971	0.3788	−1.714	−0.229	6.573	0.010
3–4	−0.981	0.5046	−1.970	0.008	3.780	0.052
1–2	0^a^	–	–	–	–	–
CPOT	0.040	0.1046	−0.165	0.245	0.147	0.702
CRP (mg/L)	0.037	0.0166	0.005	0.070	5.003	0.025
TNFα (pg/ml)	0.007	0.0046	−0.002	0.016	2.316	0.128
IL−6 (pg/ml)	0.000	0.0016	−0.003	0.003	0.003	0.955
Sleep duration (min)	−0.012	0.0094	−0.030	0.006	1.629	0.202

TBI, traumatic brain injury; GSES, general self-efficacy scale; CPOT, critical-care pain observation tool; AIS, Athens insomnia scale; CRP, C-reactive protein; TNF α, tumor necrosis factor α; IL-6, Interleukin-6.

0^a^ refers to the redundant parameter; β refers to the regression coefficient or model parameter; “–” indicates that the group is the reference group.

### 3.4 Comparison of HADS-A scores between delirium and non-delirium patients with TBI

**Figure 2:**
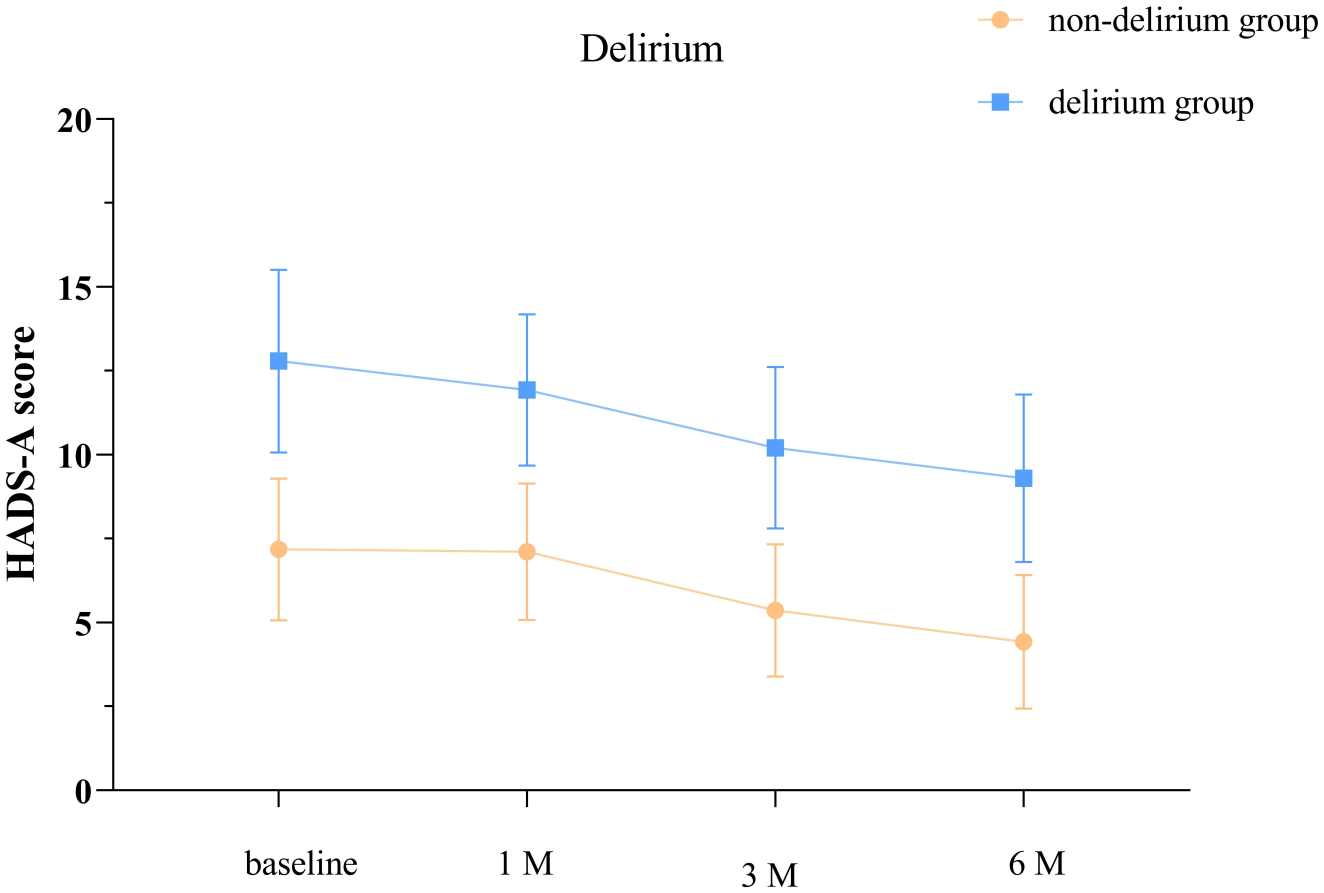
Anxiety scores at different time points in the delirium and non-delirium groups. The baseline refers to the day of discharge. HADS-A: hospital anxiety and depression scale- anxiety score. The error bars indicate the standard deviation, the larger the error bars, the greater the variability or uncertainty in the data.

According to the separate effects analysis of the GEE model, significant differences in HADS-A scores were observed between delirium and non-delirium groups on discharge day, at one, three, and 6 months post-discharge (*P* < 0.001, Supplementary Table S1; Figure 2). Compared to discharge day, non-delirium patients showed significant differences in HADS-A scores at 3 and 6 months post-discharge (*P* < 0.05), while delirium patients exhibited significant differences at 1, 3, and 6 months post-discharge (*P* < 0.05).

### 3.5 Comparison of HADS-A scores among TBI patients with different sleep quality levels

**Figure 3:**
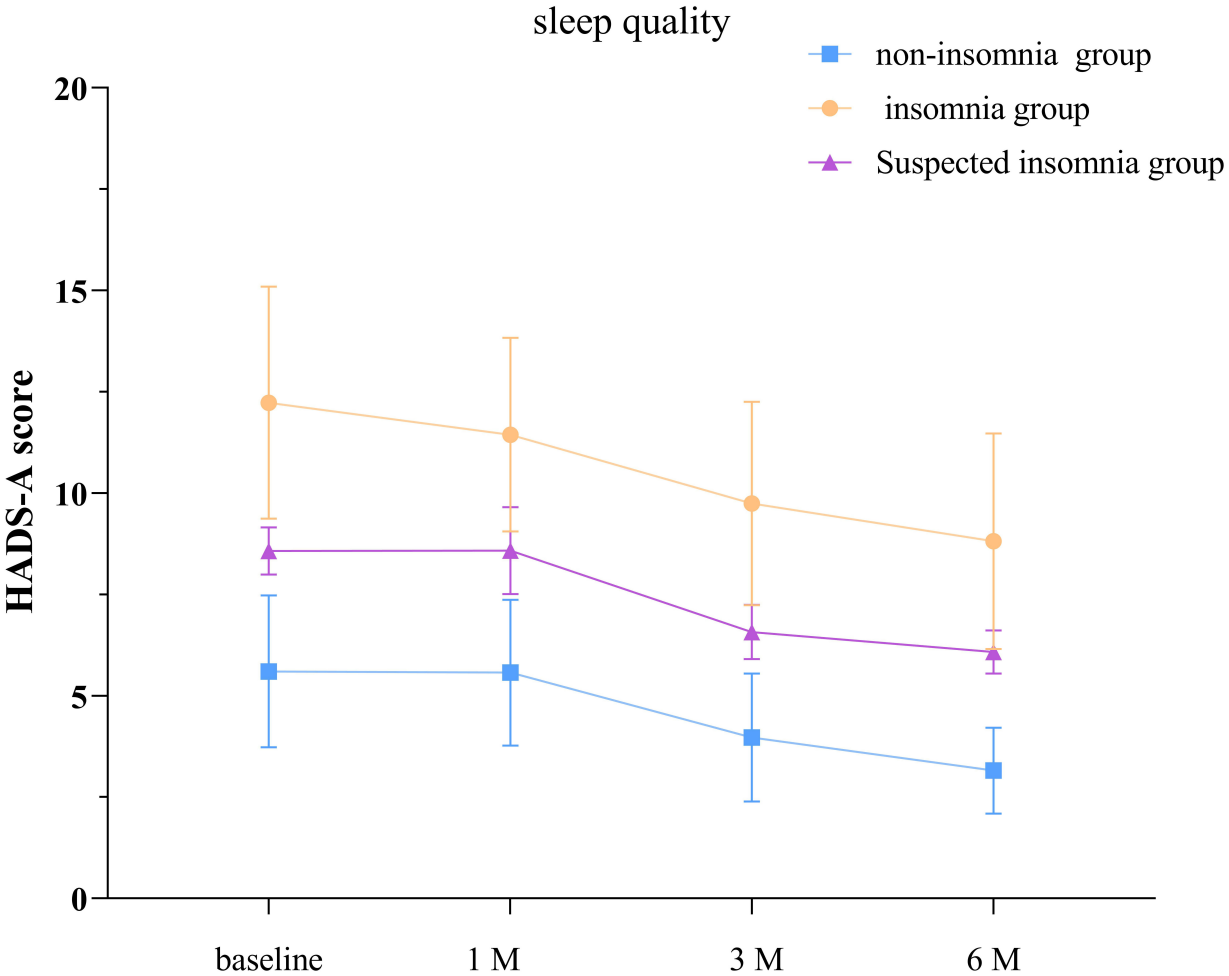
Anxiety scores in groups with different sleep quality. The baseline refers to the day of discharge. HADS-A: hospital anxiety and depression scale- anxiety score. The error bars indicate the standard deviation, the larger the error bars, the greater the variability or uncertainty in the data.

The separate effects analysis of the GEE model revealed significant differences in HADS-A scores among patients categorized into normal sleep, suspected insomnia, and insomnia groups on discharge day, as well as at 1, 3, and 6 months post-discharge (*P* < 0.001, see Supplementary Table S2; Figure 3). Compared to discharge day, patients with normal sleep exhibited significant differences in HADS-A scores at 3 and 6 months post-discharge (*P* < 0.05). Similarly, the suspected insomnia group showed significant differences at 3 and 6 months post-discharge (*P* < 0.05), while the insomnia group displayed significant differences at 1, 3, and 6 months post-discharge (*P* < 0.05).

### 3.6 Comparison of HADS-A scores among TBI patients with different levels of self-efficacy

**Figure 4:**
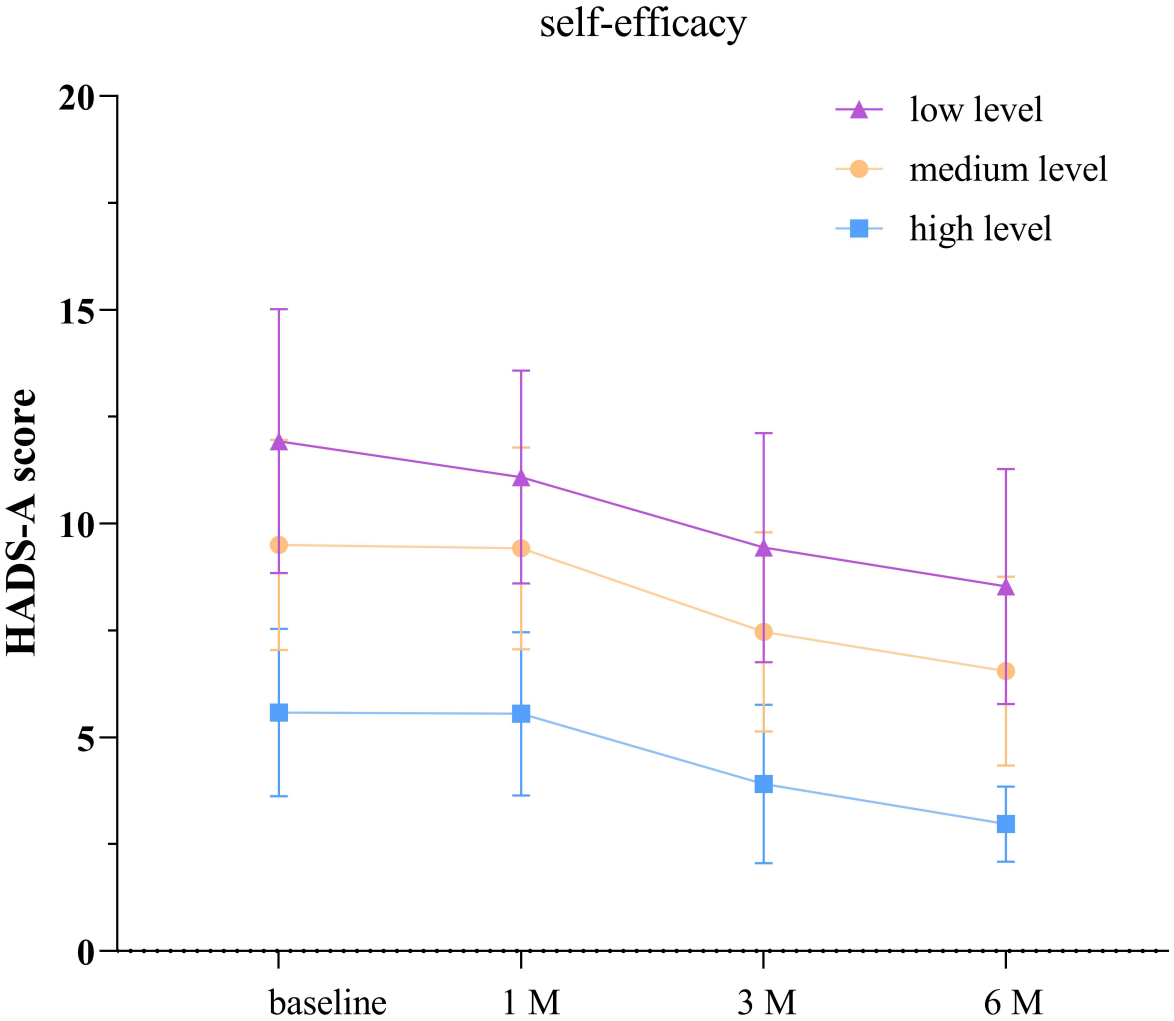
Anxiety scores in groups with different levels of self-efficacy. The baseline refers to the day of discharge. HADS-A: hospital anxiety and depression scale- anxiety score. The error bars indicate the standard deviation, the larger the error bars, the greater the variability or uncertainty in the data.

According to the separate effects analysis of the GEE model, significant differences in HADS-A scores were observed between patients with low self-efficacy and those with moderate self-efficacy, low self-efficacy and high self-efficacy, as well as moderate self-efficacy and high self-efficacy groups on discharge day, at 1, 3, and 6 months post-discharge (*P* < 0.05, see Supplementary Table S3; Figure 4). Compared to discharge day, the low self-efficacy group showed significant differences in HADS-A scores at 1, 3, and 6 months post-discharge (*P* < 0.05), the moderate self-efficacy group exhibited significant differences at 3 and 6 months post-discharge (*P* < 0.05), and the high self-efficacy group displayed significant differences at 3 and 6 months post-discharge (*P* < 0.05).

### 3.7 Comparison of HADS-D scores among TBI patients with different levels of self-efficacy

**Figure 5:**
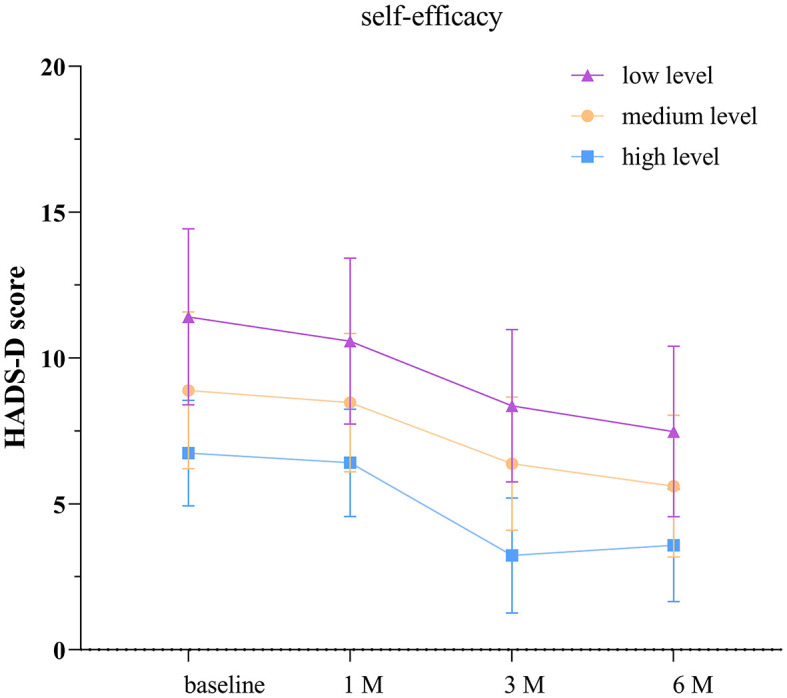
Depression scores in groups with different levels of self-efficacy. The baseline refers to the day of discharge. HADS-D: hospital anxiety and depression scale- depression score. The error bars indicate the standard deviation, the larger the error bars, the greater the variability or uncertainty in the data.

According to the separate effects analysis of the GEE model, significant differences in HADS-D scores were observed between patients with low self-efficacy and those with moderate self-efficacy, low self-efficacy and high self-efficacy, as well as moderate self-efficacy and high self-efficacy groups on discharge day, at 1, 3, and 6 months post-discharge (*P* < 0.05, see Supplementary Table S4; Figure 5). Compared to discharge day, the low self-efficacy group showed significant differences in HADS-D scores at 1, 3, and 6 months post-discharge (*P* < 0.05), the moderate self-efficacy group exhibited significant differences at 3 and 6 months post-discharge (*P* < 0.05), and the high self-efficacy group displayed significant differences at 3 and 6 months post-discharge (*P* < 0.05).

## 4 Discussion

Our study identified several key factors associated with heightened anxiety symptoms in TBI patients: younger age, underlaying disease, TBI classification, delirium, elevated CRP levels, diminished self-efficacy, and insomnia. These findings underscore the intricate interplay of biological, psychosocial, and clinical elements contributing to anxiety development post-TBI. Interestingly, depression symptoms did not correlate significantly with delirium or insomnia but exhibited significant associations with serum CRP levels, TBI classification, and self-efficacy.

### 4.1 The relationship between CRP, delirium, anxiety, and depression in patients with TBI

In this study, both HADS-A and HADS-D scores were higher in patients with delirium compared to non-delirious patients. However, only the intergroup difference in HADS-A was statistically significant. Consistent with Barker et al., anxiety symptoms were more prevalent than depression symptoms when using HADS (Barker-Collo et al., 2018). There was no statistically significant difference in HADS-D scores between the delirium and non-delirium groups, potentially explained by the majority (96.4%) of the delirium group being moderate TBI patients, who may have more severe cognitive deficits, potentially masking emotional symptoms and/or poorer insight, resulting in fewer self-reported emotional symptoms (Uiterwijk et al., 2022). The GEE analysis in this study revealed that mild TBI patients reported more severe anxiety and depression than moderate TBI patients, indirectly confirming this speculation. TBI-related delirium is a complex and poorly understood complication occurring in a heterogeneous patient population (Sherer et al., 2020). It is speculated that the pathophysiology of TBI-related delirium differs from other delirium processes (Povlishock and Katz, 2005). Even mild or moderate TBI patients may experience post-traumatic delirium without significant structural damage detected by neuroimaging (Ganau et al., 2018). The underlying pathophysiology remains incompletely understood, but severe and persistent inflammation is associated with a series of molecular, biochemical, and cellular changes in the brain that lead to neuronal injury and apoptosis (Ganau et al., 2018; Sun et al., 2019). The mechanisms underlying anxiety and depression following TBI are currently unclear, but inflammation post-TBI is speculated to be a contributing factor, associated with elevated levels of TNF-α, IL-6, IFN-α, and CRP (Malik et al., 2022; Rathbone et al., 2015).

In our study, delirious patients exhibited higher post-injury CRP levels compared to non-delirious patients, and elevated CRP levels were associated with higher anxiety and depression scores in patients. Studies have shown elevated CRP levels have also been associated with delirium (Lozano-Vicario et al., 2023; Vasunilashorn et al., 2017) and psychological disorders (Wium-Andersen et al., 2013; Bekkevold et al., 2023) even in the absence of a TBI event. Increased CRP concentrations may relate to emotional disturbances and cognitive impairments, but the mechanisms remain unclear (Su et al., 2014). Systemic inflammatory responses likely play a significant role. First, systemic inflammation activates the hypothalamic-pituitary-adrenal (HPA) axis (Murray et al., 2013) and triggers stress responses, releasing stress hormones like cortisol. These hormones help manage short-term stress, but prolonged HPA activation may lead to anxiety symptoms (Tsigos and Chrousos, 2002; Juruena et al., 2020; Haroon et al., 2012). Second, systemic inflammation may increase the conversion of tryptophan to kynurenine and quinolinic acid through the indoleamine 2,3-dioxygenase pathway, reducing serotonin production. Lower serotonin levels correlate closely with anxiety, while kynurenine and quinolinic acid may harm the central nervous system and raise anxiety risk (Christmas et al., 2011; Dantzer et al., 2008). Third, systemic inflammation activates microglia in the brain, resulting in neuroinflammation (Qin et al., 2007). Neuroinflammation contributes to the pathophysiology of anxiety and depression (Briones and Woods, 2014). Finally, systemic inflammation can increase oxidative stress, damaging neurons, disrupting neurotransmitter balance, and worsening anxiety symptoms (Dantzer et al., 2008; Fedoce et al., 2018). We measured CRP at a single time point, but its link to anxiety may reflect an early inflammatory surge that increases the risk of subsequent psychological issues. This emphasizes the necessity of identifying TBI patients at high risk for delirium and psychological problems based on early CRP concentrations.

Additionally, research has reported gender-specific associations between CRP levels and symptoms of depression and anxiety, with significant positive correlations observed only in females (Yang et al., 2020). Therefore, further research in specific populations is necessary to explore the correlation between CRP levels in TBI patients and delirium, anxiety, and depression.

### 4.2 The relationship between insomnia, anxiety, and depression in patients with TBI

Our study indicates a close association between insomnia and anxiety symptoms following TBI, while its connection with depression remains unclear. In the general population, sleep issues and mental health problems often co-occur bidirectionally, a well-established phenomenon (Scott et al., 2021). However, in the context of TBI, poor sleep may be influenced by various mechanisms including severity of injury (Fichtenberg et al., 2000; Mahmood et al., 2004), location of injury (Leduc et al., 2007), post-injury physiological factors (e.g., biochemical changes due to injury) (Mathias and Alvaro, 2012), alterations in melatonin levels (Grima et al., 2016), and psychological factors (Fogelberg et al., 2012). These factors could potentially impact our study outcomes. Previous studies have shown that subjective poor sleep quality and insomnia in TBI patients are significantly associated with increased levels of depression and/or anxiety (Huang et al., 2013; Johnson et al., 2019). However, these associations were not consistently reported when objectively measuring sleep quality (El-Khatib et al., 2019; Botchway et al., 2019). These findings suggest inconsistency in objectively reporting sleep quality in relation to depression and anxiety. Future research in this population should include longitudinal tracking of sleep in TBI patients to understand changes in subjective and objective sleep quality and their relationship with psychopathology. Prompt identification of insomnia patients is essential to improve sleep quality and reduce anxiety. Timely identification of insomnia patients is essential to improve sleep quality and reduce anxiety.

### 4.3 The relationship between self-efficacy and anxiety/depression in patients with TBI

Higher levels of self-efficacy are negatively correlated with anxiety and depression. Enhancing self-efficacy in TBI patients may effectively improve their mental health in clinical practice. Consistent with previous research findings, patients with higher self-efficacy experience fewer emotional disturbances and enjoy a higher quality of life (Brands et al., 2015). The common-sense model of self-regulation suggests that when facing health threats, individuals navigate emotional responses to the threat, develop perceptions about the threat and potential therapeutic actions, formulate action plans to cope with the threat, and integrate continuous feedback on the effectiveness of their action plans and the progression of the threat (Leventhal et al., 2016). Other models of adaptation following acquired brain injury also emphasize the ongoing interaction between behavior and emotions, as well as the critical role of self-efficacy and coping in disease management (Brands et al., 2012). Brands et al. (2014) demonstrated that high self-efficacy in managing symptoms related to brain injury can prevent negative impacts of emotion-focused coping. This suggests that enhancing self-efficacy in patients may be a potential strategy for managing mental health issues following TBI. Research has already indicated that self-efficacy and quality of life improve following neuropsychological rehabilitation (Brands et al., 2017; Belanger et al., 2020). Hawley et al. (2022) employed a self-advocacy independent living (SAIL) program to enhance self-efficacy in TBI patients.

Future therapeutic approaches for anxiety and depression in TBI patients should explicitly focus on reinforcing patient self-belief to enhance self-efficacy.

### 4.4 Limitations

Despite revealing complex relationships between various factors and symptoms like anxiety, depression, delirium, sleep, and self-efficacy among TBI patients, our study has several limitations that must be considered. Firstly, the small sample size and short follow-up period may not capture all potential influencing factors, and selection bias among patients could affect the generalizability of the results. Secondly, identifying the independent impact of delirium still poses challenges, as this condition typically results from the interaction of multiple pathological factors (Maldonado, 2018). Thirdly, a significant proportion of the included population is male. Future research should aim to include more female participants to better represent the TBI population. Additionally, in this study, we only measured CRP, TNF-α, and IL-6 levels on day 1 post-injury. Future research should dynamically monitor serum inflammatory factors at multiple time points to clarify their effects on anxiety and depression. Given that only three mild TBI patients showed delirium, we could not perform subgroup analyses based on TBI classification. Therefore, it is unclear if the observed links between delirium and pain, sleep quality, and self-efficacy are due to differences in TBI classification. Lastly, approximately half of the patients in our study had comorbidities such as hypertension and diabetes, which could be a factor influencing cytokine levels in the studied patient group (Feng et al., 2022; Wang et al., 2024). Future studies could further expand our findings by validating them through larger-scale randomized controlled trials and delving deeper into the exact mechanisms of inflammation response in mental health issues post-TBI. Additionally, long-term follow-up studies could assess the prolonged impacts of these factors on the long-term mental health outcomes of TBI patients.

## 5 Conclusion

Our study identified significant correlations between younger age, underlying diseases, TBI classification, delirium, elevated CRP levels, lower self-efficacy, insomnia, and more severe anxiety symptoms. Conversely, there was no significant association between depression symptoms and delirium or insomnia, but significant relationships were found with serum CRP levels, TBI classification, and self-efficacy. In the future, serum CRP levels post-TBI hold promise as a biomarker to identify patients at risk for emotional symptoms. Additionally, comprehensive interventions targeting prevention and treatment of delirium, timely management of insomnia, and enhancement of patient self-efficacy could be promising strategies to improve mental health outcomes.

## Data Availability

The original contributions presented in the study are included in the article/Supplementary material, further inquiries can be directed to the corresponding author.
