# Supplementary material for: Analysis of the correlation and influencing factors between delirium, sleep, self-efficacy, anxiety, and depression in patients with traumatic brain injury: a cohort study
Source: Front Neurosci. 2024 Nov 1;18:1484777. doi: 10.3389/fnins.2024.1484777 (PMC11564178; doi:10.3389/fnins.2024.1484777)
Supplement: Supplementary file 1 [file Data_Sheet_1.docx]

| **Table S1 Comparison of HADS-A score between the delirium and non-delirium groups** | | | |  |
| --- | --- | --- | --- | --- |
| Time | non- delirium group  mean (SD)  (n=71) | delirium group  mean (SD)  (n=56) | Between group  differences (*95%CI*) | *P value* |
| baseline | 7.18±2.11 | 12.79±2.72 | 5.60 (4.74, 6.46) | <0.001 |
| 1 month | 7.11±2.03 | 11.93±2.25* | 4.82(4.07,5.56) | <0.001 |
| 3 months | 5.36±1.97* | 10.21±2.41* | 4.85(4.05,5.52) | <0.001 |
| 6 months | 4.43±1.99* | 9.30±2.49* | 4.87(4.07,5.69) | <0.001 |

HADS-A: hospital anxiety and depression scale - anxiety subscale, SD: standard deviation, CI: confidence interval

* indicates statistically significant difference compared to the discharge day (*P* < 0.05).
