# Supplementary material for: Analysis of the correlation and influencing factors between delirium, sleep, self-efficacy, anxiety, and depression in patients with traumatic brain injury: a cohort study
Source: Front Neurosci. 2024 Nov 1;18:1484777. doi: 10.3389/fnins.2024.1484777 (PMC11564178; doi:10.3389/fnins.2024.1484777)
Supplement: Supplementary file 2 [file Data_Sheet_2.docx]

| **Table S2 Comparison of HADS-A score according to the sleep** | | | | | | |
| --- | --- | --- | --- | --- | --- | --- |
| Time | non-insomnia mean (SD)  (n=35) | suspected insomnia  mean (SD)  (n=26) | insomnia mean (SD)  (n=66) | *P value*  *(A vs. B)* | *P value*  *(A vs. C)* | *P value*  *(B vs. C)* |
| baseline | 5.60(1.87) | 8.57(0.58) | 12.23(2.86) | <0.001 | <0.001 | <0.001 |
| 1 month | 5.57(1.80) | 8.58(1.07) | 11.44(2.39)* | <0.001 | <0.001 | <0.001 |
| 3 months | 3.97(1.58)* | 6.57(0.67)* | 9.74(2.51)* | <0.001 | <0.001 | <0.001 |
| 6 months | 3.15(1.06)* | 6.08(0.53)* | 8.81(2.66)* | <0.001 | <0.001 | <0.001 |

HADS-A: hospital anxiety and depression scale - anxiety subscale, SD: standard deviation

A: non-insomnia group, B: suspected insomnia group, C: insomnia group

* indicates statistically significant difference compared to the discharge day (*P* < 0.05).
