# Supplementary material for: Analysis of the correlation and influencing factors between delirium, sleep, self-efficacy, anxiety, and depression in patients with traumatic brain injury: a cohort study
Source: Front Neurosci. 2024 Nov 1;18:1484777. doi: 10.3389/fnins.2024.1484777 (PMC11564178; doi:10.3389/fnins.2024.1484777)
Supplement: Supplementary file 3 [file Data_Sheet_3.docx]

| **Table S3. Comparison of HADS-A score according to the self-efficacy level** | | | | |  |  |
| --- | --- | --- | --- | --- | --- | --- |
| Time | low level  mean (SD)  (n=58) | medium level mean (SD)  (n=38) | high level mean (SD)  (n=31) | *P value*  *low vs. medium* | *P value*  *low vs. high* | *P value*  *medium vs. high* |
| baseline | 11.93(3.09) | 9.50(2.46) | 5.58(1.96) | <0.001 | <0.001 | <0.001 |
| 1 month | 11.09(2.49)* | 9.42(2.36) | 5.55(1.91) | 0.014 | <0.001 | <0.001 |
| 3 months | 9.44(2.68)* | 7.47(2.33)* | 3.91(1.85)* | <0.001 | <0.001 | <0.001 |
| 6 months | 8.53(2.75)* | 6.55(2.21)* | 2.97(0.88)* | <0.001 | <0.001 | <0.001 |

HADS-A: hospital anxiety and depression scale - anxiety subscale, SD: standard deviation

* indicates statistically significant difference compared to the discharge day (*P* < 0.05).
