# Supplementary material for: Analysis of the correlation and influencing factors between delirium, sleep, self-efficacy, anxiety, and depression in patients with traumatic brain injury: a cohort study
Source: Front Neurosci. 2024 Nov 1;18:1484777. doi: 10.3389/fnins.2024.1484777 (PMC11564178; doi:10.3389/fnins.2024.1484777)
Supplement: Supplementary file 4 [file Data_Sheet_4.docx]

| **Table S4. Comparison of HADS-D score according to the self-efficacy level** | | | | |  |  |
| --- | --- | --- | --- | --- | --- | --- |
| Time | low level  mean (SD)  (n=58) | medium level  mean (SD)  (n=38) | high level  mean (SD)  (n=31) | *P value*  *low vs. medium* | *P value*  *low vs. high* | *P value*  *medium vs. high* |
| baseline | 11.41(3.02) | 8.89(2.69) | 6.74(1.81) | <0.001 | <0.001 | <0.001 |
| 1 month | 10.58(2.84)* | 8.47(2.37) | 6.41(1.84) | <0.001 | <0.001 | <0.001 |
| 3 months | 8.36(2.61)* | 6.38(2.28)* | 3.23(1.97)* | <0.001 | <0.001 | <0.001 |
| 6 months | 7.48(2.92)* | 5.61(2.43)* | 3.58(1.93)* | 0.022 | <0.001 | <0.001 |

HADS-D: hospital anxiety and depression scale - depression subscale, SD: standard deviation

* indicates statistically significant difference compared to the discharge day (*P* < 0.05).
